# Light People: Professor Fan Wang

**DOI:** 10.1038/s41377-023-01263-7

**Published:** 2023-09-01

**Authors:** Siqiu Guo

**Affiliations:** Light Publishing Group, Changchun Institute of Optics, Fine, Mechanics and Physics, Chinese Academy of Sciences, Changchun, China

## Abstract

My first encounter with Prof. Fan Wang left a profound impression on me. I felt that he was exactly the gentle and courteous scholar depicted in books, well-read in poetry and literature, and exceptionally talented. Through my interactions with Prof. Fan Wang, I deeply sensed his passion for academia and pursuit of knowledge, as well as his warm hospitality, kindness, and gentle demeanor.

His thinking is profound and broad, capable of examining issues from various perspectives, and providing inspiration. Prof. Fan Wang is a leading young scientist who actively engages in various academic activities, concerns himself with cutting-edge technological issues, and dedicates himself to overcoming research challenges that can drive advancements and developments in optoelectronics, biophotonics and nanomaterials.

Prof. Fan Wang’s talent, knowledge, character, and sense of family responsibility all fill me with admiration and respect. Now, let’s step into the world of Light People Prof. Fan Wang and together, appreciate the brilliance of his carefree and extraordinary life.

**Figure:**
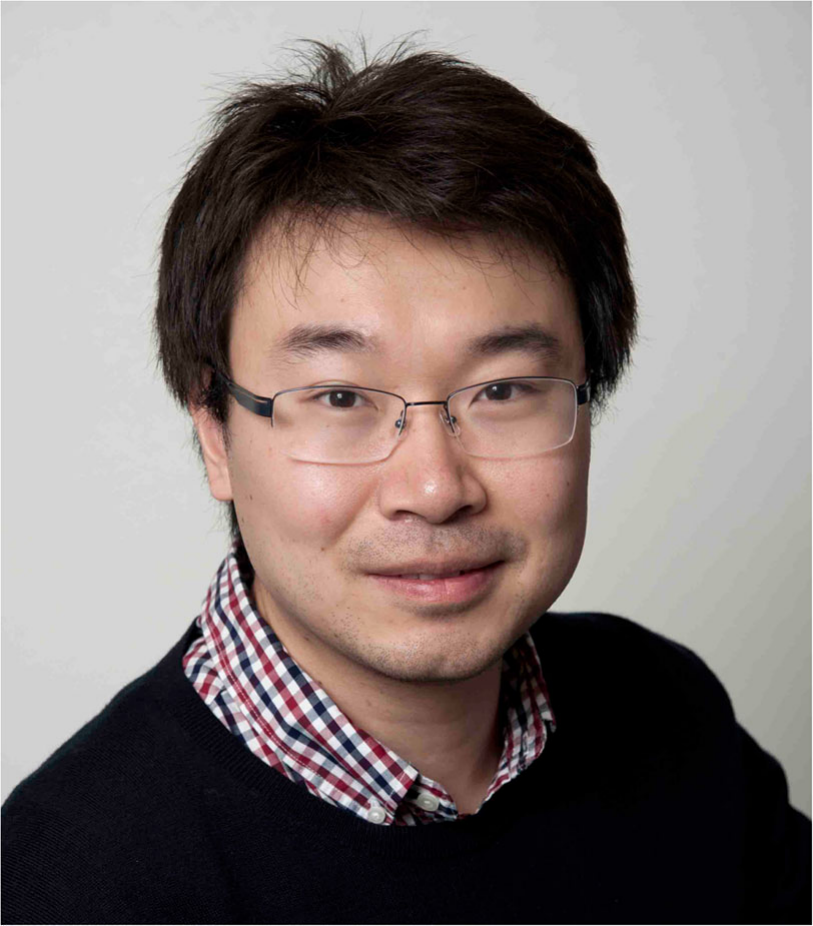

**Short Bio:** Prof. Fan Wang leads the biophotonics research group at the School of Physics, Beihang University. Prof. Wang has expertise in optoelectronics, biophotonics, and nanomaterials. He also has expertise in the biophotonics application of nanomaterials, including super-resolution microscopy, optical tweezers, single nanoparticle tracking and sensing. Prof. Wang has published over 80 peer-reviewed journal articles (including 13 *Nature* series journals), including leading author articles in *Nature Nanotechnology, Light: Science & Applications, Nature Communications* x2, *Optica, Nano Letters* x6, *Small, Advanced Science* and *Advanced Materials*, with an h-index of 36 and over 5000 citations. Prof. Wang obtained his PhD from the University of New South Wales in 2014. In 2019, he was awarded the UTS Chancellor’s Postdoctoral Research Fellowship to establish his biophotonics research team. In 2020, he obtained the Australia Discovery Early Career Researcher Award to conduct his biological laser cooling technology research, and he joined the School of Electrical and Data Engineering to establish his group. In 2021, he was awarded the David Syme research prize and iCANX Young Scientist Awards due to his biophotonics research. In 2022, he joined Beihang University to establish his group in China.

**1. During the past decade of studying and working in Australia, you have achieved numerous remarkable scientific research accomplishments, earning you prestigious honors such as the Australian Young Scholar Award, the Australian David Syme Research Award and the iCANX Young Scientist Award. Could you please briefly introduce your groundbreaking achievements in the fields of super-resolution microscopic imaging and optical tweezers technology?**

The achievements made over the years are the result of the team’s collective efforts, and the students all displayed tremendous dedication. Overall, it can be considered a commendable accomplishment in the field of interdisciplinary studies. With my diverse interests, I delved into various subjects and eventually focused my main efforts on interdisciplinary research. I often told my students that our optical research must encompass the characteristics and advantages of materials, while our material research should aim to apply optical technology. Learning from the experiences of others can help us make progress in our own fields, which is the essence of interdisciplinary studies.

I started to know super-resolution imaging in 2017 when I officially joined the research team led by Academician Dayong Jin. Initially, I assisted him in doing some related work published in *Nature*^1^, which sparked my strong interest in various super-resolution techniques. These techniques, like the unique attributes of ancient Chinese generals in the game “Three Kingdoms”, all had their distinct features, yet they shared the common limitation of insufficient imaging depth. Most super-resolution techniques were limited to imaging single cells, and achieving in vivo super-resolution imaging remained a challenge. Previously, Prof. Martin Booth from the University of Oxford adopted an adaptive compensation method, while I innovatively combined the advantages of near-infrared fluorescence imaging using nanomaterials from Prof. Jin’s research group to develop a series of techniques suitable for deep tissue imaging. These techniques were based on the unique nonlinear response properties of upconversion nanomaterials and showed excellent results. A notable example is in 2018 when, based on the fluorescence saturation property of the material, we used a vortex beam to penetrate a 100 μm deep biological tissue, achieving imaging with a resolution of 50 nm^2^. This breakthrough can be likened to seeing a busy ant’s leg hair clearly through smoke with naked eyes. This work provided a milestone value and has been cited over 150 times. Subsequently, we further conducted a series of studies using the superlinear fluorescence effect and differences between energy levels in upconversion particles. Additionally, taking inspiration from Prof. Peng Xi’s paper entitled “Mirror-enhanced super-resolution microscopy” which was published in *Light: Science & Applications (Light)*^3^, we leveraged the advantage of multi-ion fluorescence radiation in rare-earth-doped nanomaterials to achieve fluorescence self-interference effects in single nanomaterials. And with this effect, we developed the rapid distance imaging technology, achieving a sensing precision of 1 nm at imaging frame rates exceeding 50 Hz^4^.

During my doctoral studies in 2010, I began to conduct research in optical tweezers, but my focus was different from mainstream biological applications. Instead, my specific direction was to use optical tweezers to study the spectroscopic properties of single nanomaterials. At that time, I collaborated with Chennupati Jagadish, President of the Australian Academy of Science, became one of the early researchers using optical tweezers to study semiconductor nanowires. We developed a profound friendship through our shared interest. Conducting optical tweezers at the nanoscale presented significant challenges, as the small size resulted in a small overall polarization and optical force. This was especially true for particles with a small refractive index, such as when placing a glass bead in a glass of water, you wouldn’t be able to see the bead due to minimal refraction. In other words, the optical force will be too weak. As a result, gold particles were commonly used as probes at the nanoscale, but they had the drawback of significant heating. However, when using upconversion particles for optical tweezers, we discovered something fascinating: These particles had an extremely low refractive index, but their manipulation was remarkably stable. This prompted us to develop a new technology to investigate the reason behind this stability. The world is indeed marvelous, and we found that it was the result of resonance from thousands of ions within the particle. This resonance directly maximized the polarization, forming the most powerful optical force probe at the nanoscale^5^. Moreover, these particles could simultaneously act as “optical force dyes”, enabling us to stain different locations and organelles in cells. While traditional dyes would only allow imaging where they were applied, these “optical force dyes” could enhance the optical force during imaging to facilitate effective manipulation.

**2. You have had three postdoctoral experiences in Australia. Can you share with us your extraordinary experiences during this period?**

During my time in Australia, I changed schools quite a bit, having spent time at almost all the universities in Sydney, including the University of New South Wales, the University of Sydney, Macquarie University, and the University of Technology Sydney (UTS), in addition to the Australian National University in Canberra. Five universities, five diverse experiences. But this experience was not that extraordinary for researchers like us. Before securing a tenure position, it is common to move around and explore different places. As I had a strong affinity for Australia, I chose to stay here.

My postdoctoral experience can be summarized in these words: Building labs and being seniors. Each lab had its legends about the seniors, as they were often the first to set up the labs. They had to work without guidance, making their journey more challenging. Their time was torn apart by various issues, scattered on the ground like fragments. But later on, these experiences became the foundation of the labs. And of course, they underwent more challenges than others.

My first postdoctoral experience was actually during the last year of my PhD. It was almost like being a postdoc without the formal title. I was the first PhD student of my PhD supervisor, Peter Reece. During the first two years of my PhD, I was the only PhD student in the group. Peter Reece had just returned to the University of New South Wales, and initially, we have to set up various systems and debugging various equipment. Only later did we have time for actual research. The last year of my PhD and my postdoctoral experience were quite similar, as I had to set the research direction, maintain equipment, and guide junior students. At that time, I collaborated frequently with Academician Chennupati Jagadish, who was also very kind. There was an urgent need for optical characterization on their side, so even before submitting my thesis in 2013, I went to work as a postdoc with him. This change led me from optical tweezers to nanophotonics.

Those two years were relatively relaxing, and time seemed to stretch endlessly. It was like magic. Two years seemed to be more than a couple of years, and I felt like I had plenty of time every day, even though I was very busy. As the senior, I supervised many PhD students and collaborators, designing and guiding photonics experiments, and rebuilding several sets of micro time-resolved spectroscopy systems and photocurrent systems, all while maintaining and training commercial systems. I just didn’t have much time for my own research. In the past, time passed slowly, leaving room for communication and helping others. That’s why some friends and collaborators have been working together with me till now.

Later on, when considering signing a contract for a longer duration of time, Prof. Dayong Jin, who also headed the Light Sydney Office, promised me the moon of biophotonics. And I still dream about the moon from time to time. As a result, my family and I moved back to Sydney, and I joined the newly established ARC Centre of Excellence for Nanoscale BioPhotonics at Macquarie University, intent on spending the next seven years or even longer there. We even bought a house nearby. However, such stability doesn’t quite fit what a normal researcher has to do. Not long after, Academician Jin moved to the UTS, and due to experimental resources, I began working simultaneously with Prof. Jim Piper (It is with great sadness that Emeritus Professor James (Jim) Piper passed away on July 20th, 2023) at Macquarie University and Academician Jin’s team at the UTS. Juggling both sides made time compress like a pulse compression machine that suddenly shortened it. I became extremely busy every day, especially at the UTS, where I was the first postdoc that Prof. Jin brought in. I was also the only postdoc in the field of optics, responsible for managing the direction of optical research and building the lab from scratch. This time, I became a super senior.

Setting up equipment in two universities was indeed very challenging, so I soon became a full-time postdoc at the UTS to start my third postdoc. As mentioned earlier, the benefit of time compression is that the output peak becomes higher, but the output frequency slows down. With full-time work, I had more students to oversee, and the output frequency increased. Pumped by the high-intensity work, my academic career was finally galvanized. After some twists and turns, I obtained the Chancellor’s Postdoctoral Research Fellow of the UTS and the ARC Discovery Early Career Researcher Award, marking the end of my postdoctoral journey. Following this, in 2020, I secured a lecturer position at the School of Electrical and Data Engineering at the UTS and started building my own lab from scratch again. Of course, at that time, I didn’t know that two years later, I would need to rebuild the lab once again.

**Figure:**
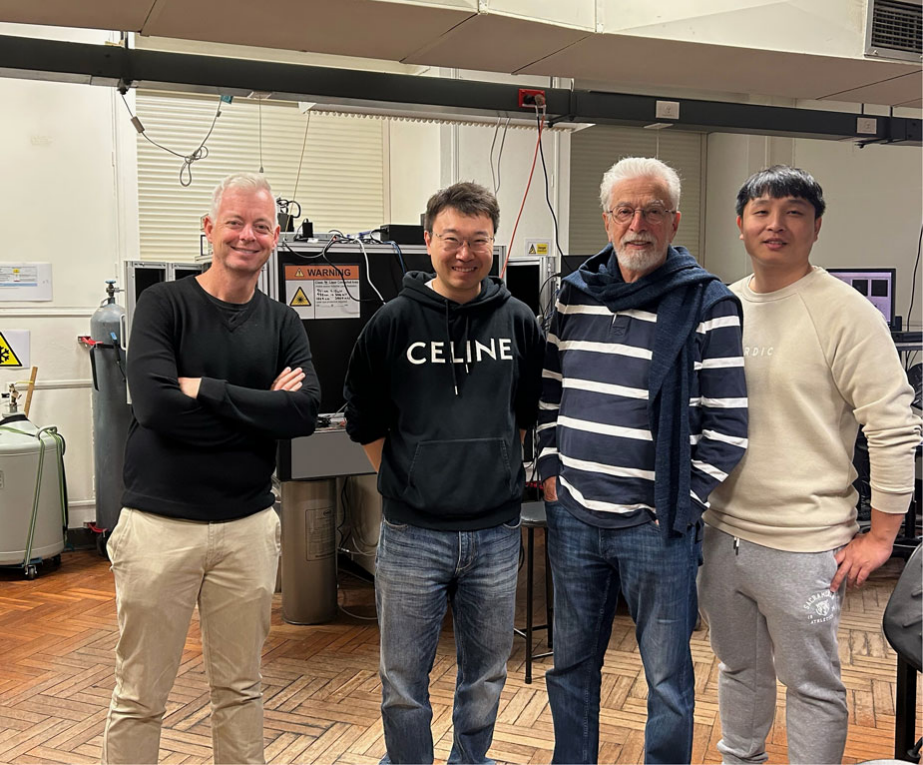
Four generations together. Peter Reece- Prof. Fan Wang’s PhD advisor, Prof. Fan Wang, Michael Gal- Peter Reece’s advisor, and Dr. Zhaohao Chen- Prof. Fan Wang’s former PhD student (from left to right)

**Figure:**
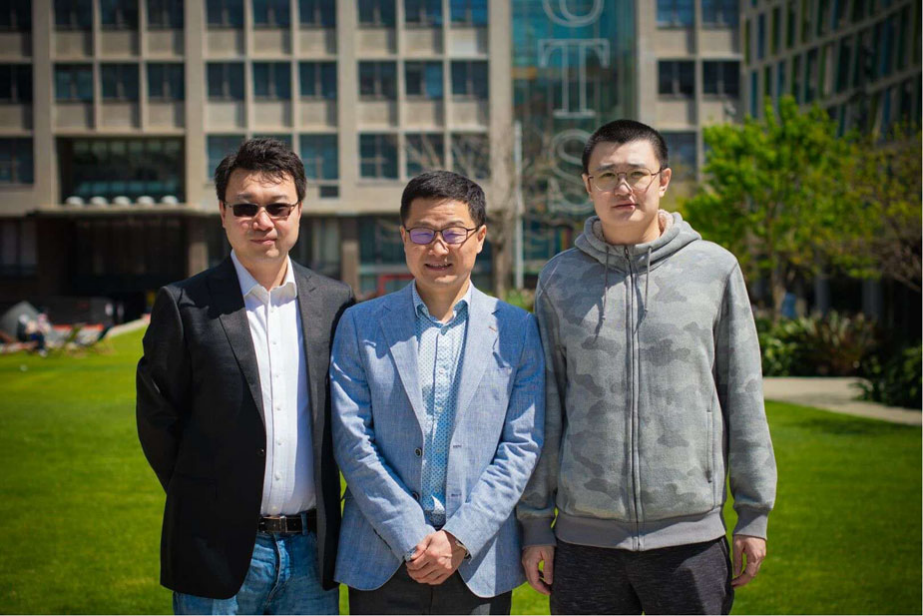
Prof. Fan Wang, Academician Dayong Jin, and Dr. Xuchen Shan- Prof. Fan Wang’s former PhD student (from left to right)

**Figure:**
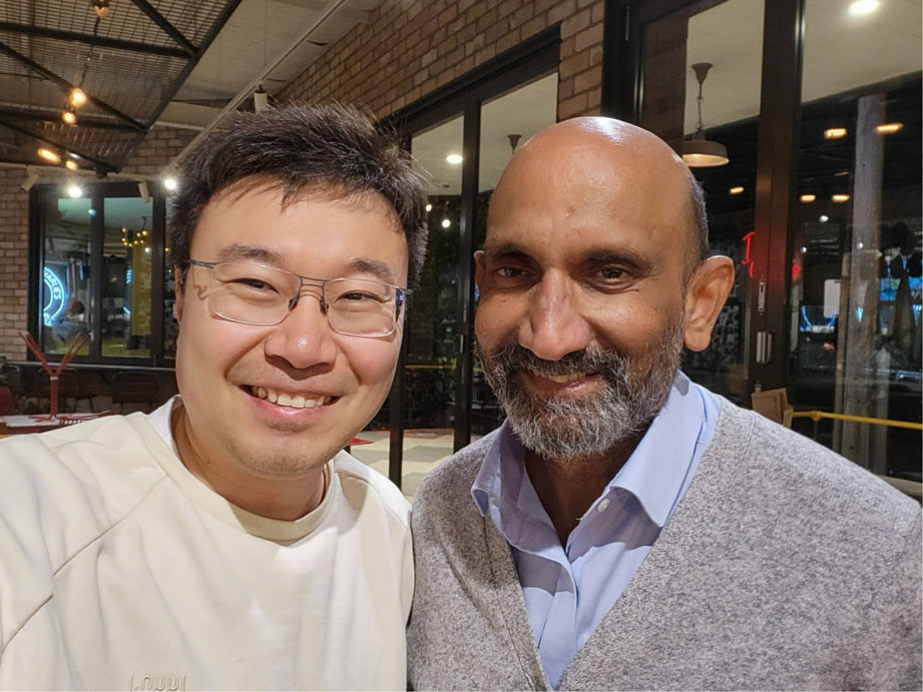
Prof. Fan Wang and Academician Chennupati Jagadish

**3. In 2022, you returned to your homeland with dreams and established your own research team. Please introduce your research direction and share the latest progress in your research?**

In 2022, I returned to Beihang University in Beijing as an overseas young talent and rejoined the Department of Applied Physics, where I had completed my undergraduate studies. I saw my former teachers and felt nostalgic when I observed the students, who reminded me of myself 20 years ago. But I had to start building my lab from scratch again. It took more than a year, and I primarily relied on my previous two PhD students, Dr. Shan Xuchen and Dr. Liu Baolei, who are now lecturers at Beihang University and the seniors in our lab. Considering the resources, connections and my interests after coming back, I took advantage of my strengths and avoided my weaknesses to blaze a trail. The research direction in my lab now revolves around three main areas.

The first direction involves continuing the research in optical tweezers with nanoprobes. Our previous research discovered the world’s most potent optical force probes, and now is the best time to follow up. Just recently, we used upconversion nanoparticles to develop a super-resolution photonic force microscope, which, for the first time, has achieved ultra-weak force measurements in three-dimensional space in an aqueous solution. It has reached the thermal limit of nanoscale force measurements in theory and significantly improved the measurement time for forces at the subfemtonewton level, approximately 50 times faster than previously reported. This technology would play a significant role in life sciences, as it would not only enable the study of antigen-antibody binding, DNA base pair binding, weak responses of cell surface integrin proteins, but help observe dynamic mechanical responses. Several other projects in this direction are currently being carried out under the supervision of Dr. Shan Xuchen.

The second direction involves super-resolution imaging and computational optics, which is one of the hotspots in modern optical development. Building upon the previous use of nonlinear enhancement with probes, our recent work involves using dynamic stepwise algorithms to solve nonlinear changes in upconversion fluorescence images, thus enhancing the image resolution. The advantage of this method is that it can be combined with various imaging modes to achieve multimodal gain. In the field of computational optics, we extended the previously developed self-evolving ghost imaging technology to different wavelengths and imaging modes, overseen by Dr. Liu Baolei.

The third direction is the one that I have been most interested in and personally responsible for, which is the integration of super-resolution, computational optics, and characterization and photonic manipulation of low-dimensional materials. But at this stage, I only have some preliminary ideas, so I am researching it on my own. Just like watching TV dramas or talk shows, following the routine makes it predictable, and knowing what will happen next loses its appeal. Of course, I’m now stretched thin. So starting a new research direction would be much more challenging than before.

**4. In 2018, your paper titled “Microscopic inspection and tracking of single upconversion nanoparticles in living cells” published in** ***Light***^6^**, was selected as the cover paper for issue 3. Could you please introduce the innovations in this paper and share the story behind the cover image?**

It was during my third postdoctoral period in 2017 that I worked on this paper, which represents a further exploration of the properties and applications of upconversion nanoparticles. While working on previous Nature articles, I built a single-particle characterization system and found that our particles had extremely strong and uniform absolute brightness, stable and controllable photon number output, and strong nonlinearity. Combining the compensation algorithm for single-particle response provided a perfect solution to the challenges in distinguishing single particles and dealing with low signal-to-noise ratios in single-particle tracking. Not only did we not need to use highly sensitive devices like EMCCD, but we also added the dimension of light intensity control for multiplexing tracing. Shortly after the publication of this paper, Nobel laureate Prof. Steven Chu visited Australia and chatted with me. He also shared a related paper that their team had been working on, which was later published in *Nature Photonics*. While conducting this work, in order to demonstrate the sensitivity and uniqueness of this method, we carried out an interesting experiment, where we invited many people from our school to identify how many photons would be needed to distinguish images with human eyes and how many photons would be needed for the cone cells to dominate color perception. Everyone was eager to participate in the experiment, and the results were excellent. This is why the cover image shows an eye.

But whose eye is it? Naturally, I had to find an eye with copyright permission. At the time of designing the cover, I was attending a conference abroad and had even lost my laptop. I bought a new laptop to install software for making this image. Originally, I intended to create a design with modeling, but it felt awkward no matter how I looked at it. Then, I turned my head and saw my wife. I love my wife’s eyes, so I just took a photo and used it directly, and the effect turned out to be quite good. Later on, I would sometimes use my wife’s photos for some occasions, which made her even more supportive of my work.

**Figure:**
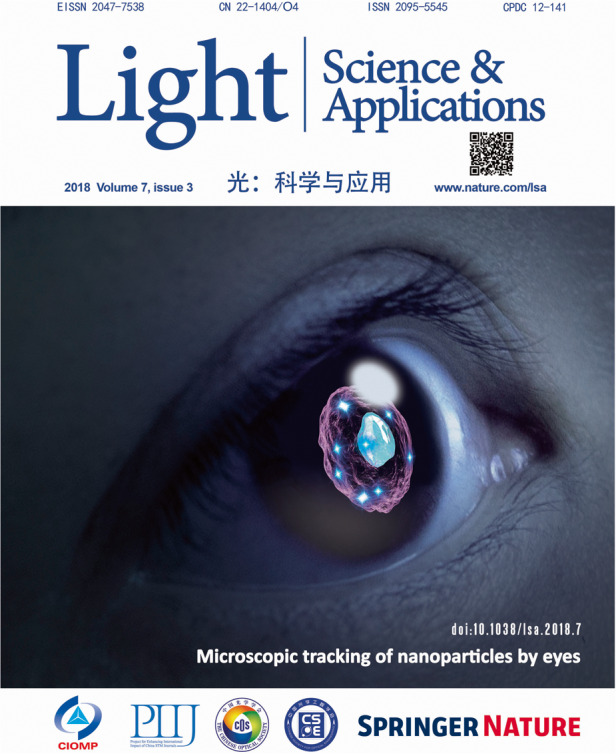
The cover of LSA’s 2018 Volume 7, Issue 3

**Figure:**
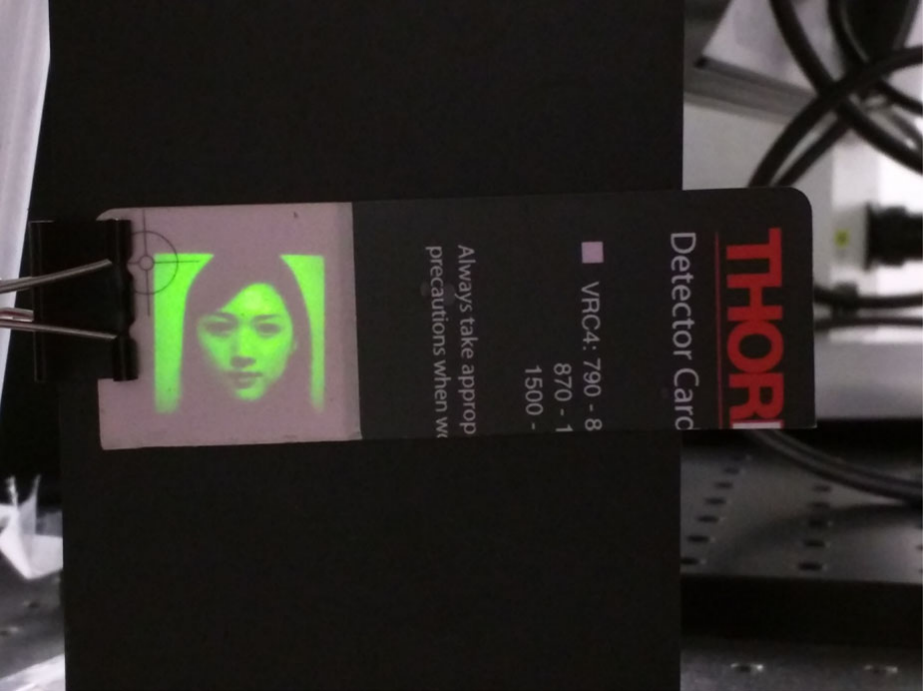
My wife’s image in the experiment: The modulation of an acousto-optic modulator, using a laser to draw a grayscale image on a fluorescence card

**5. You place great importance on promoting effective transformation and application of relevant technological. In your opinion, what are the future trends in the commercialization of optical super-resolution technology?**

Technology commercialization is an essential part of scientific research, as it allows us to see that those ideas once confined to our minds can blossom into valuable results. In China, the environment for technology commercialization is particularly favorable. Currently, there are numerous manufacturers producing super-resolution microscopy devices, each with its unique features. But mainstream devices are still relatively large in size. So the trend in development is towards miniaturization, even chip-based or modularized super-resolution microscopy. In scientific research, many research groups have used micro-nanofabrication techniques to transplant techniques such as STORM, SIM, frequency-shifted super-resolution, dark-field microscopy, and differential interference contrast microscopy onto chips. By combining these chips with ordinary microscopes, super-resolution and other modalities can be achieved. If these technologies can provide stable and continuous image outputs, their commercial prospects are quite promising. Currently, there is also a trend towards modularization in microscopy systems. For example, the super-resolution function is offered as an independent module that can be added to traditional microscopes. We’re also intent on going for this direction, as we have some ideas, but lack sufficient manpower. So we welcome like-minded postdoctoral researchers to join our team (www.fanwanglab.com) to jointly tackle challenges.

**6. In your career, have there been any individuals or events that have had a significant impact on you? In what way?**

This is actually a very difficult question to answer because throughout my career, many people and experiences have had an impact on me. Some impacts are obvious, while others have been subtle and imperceptible, like the butterfly effect that helped me decide which direction to pursue in university. For example, in terms of attitude towards work, my PhD advisor- Peter Reece, and my mentor- Mike Gal, both from different backgrounds - one Australian and the other Hungarian - showed me how joyful research can be. Even with the burden of teaching three courses simultaneously, Peter Reece would still find time to do experiments until late at night. They made me feel the pleasure and charm of research, which provided a sense of freedom without pressure, just like Rufi’s Nika from One Piece. Their impact on me has been significant, and it has formed the basis of my philosophy when leading a team, which was to reduce pressure and allow freedom of expression. But for most people, pressure and motivation go hand in hand. The junior students, for instance, didn’t have much pressure or motivation, and as a result, they didn’t publish many papers. Traditionally, one might assume that this would limit their future development, but in reality, they all led fulfilling and diverse lives. Like rivers flowing into the sea, some are majestic and turbulent, while others meander around picturesque landscapes, and some flow gently like babbling brooks, all of them unique and splendid. It doesn’t matter whether one does the best in research or whether one continues research later on. What truly matters is whether new research ideas can ignite your passion, and whether you have the determination to spend days bending down to calibrate optical paths. These are the lessons that I learned from my teacher and good friend, Peter Reece.

**7. What abilities do you prioritize in cultivating students in your teaching? What management strategies do you employ in team building?**

For undergraduate students, the most important thing is to cultivate an interest in science and let them fall in love with research. As for master’s students, it’s important to develop a solid foundation in research and good research habits, such as setting up optical systems and programming, and most importantly, to cultivate critical thinking and the habit of seeing projects through from beginning to end. As for PhD students, the first thing to cultivate is critical thinking, which is to look at problems from a developmental perspective and not blindly believe those in authority. Only through this can you discover what others cannot. A PhD student should also develop the ability to control projects, have a broad view of research development, and be sensitive to its direction. One must understand the kind of “giant machine” we are driving with gears, how to drive it, and where it is heading.

My management strategy is quite conventional, just like most teams. I believe management is a typical pyramid structure, which is relatively efficient, where each level is responsible for their respective areas. But solely following this approach is not enough. I’ve realized that I have had too little interaction with students, so I have been constantly striving to increase the time spent interacting with them. It is also essential to grant students and team members a certain degree of freedom since everyone has their own styles. Respecting individual choices while considering the team’s development and resource balance is what I would call “adaptive” management borrowed from optics terminology. Everyone’s goals are changing, and people’s attitudes are evolving, so constant adjustments are necessary. The key is to find feedback, which comes from both short and long-term research output.

**8. How do you balance your work and family life?**

Balancing research and family life is a challenge for most researchers because it’s difficult to strictly separate the two, and both require a considerable amount of time, especially for researchers who are married and have children. To address this, I have adopted a modular approach to time management, breaking various tasks into smaller segments that align with my available time. I make use of every minute and second available. I always carry three power banks for my phones and one for my laptop in my backpack, just to ensure that I can work in any suitable situation. I use fragmented time for handling minor tasks, such as writing articles or programming, while tasks that require continuous focus are done during uninterrupted periods in the evenings. With this, I can save time for sandwiched between my home time and my children’s bedtime, and even during weekends, I try to spend as much time as possible with my family. Time flies, and once children grow up, they become adults in the blink of an eye. Perhaps when they grow up, they will become even more accomplished scientists than us, so being present for our family also serves as an indirect support for our research. Additionally, I have some hobbies, like photography and music, which I indulge in for self-entertainment. These hobbies can be very effective in helping alleviate stress.

**9. I know you have two handsome sons. What are your expectations for them?**

I want them to inherit my career and become scientists, I also want them to fulfill the dreams that I haven’t achieved, such as pursuing music and art. However, my thoughts are actually not that important; what matters is how they feel about it. My expectation is for them to be able to pursue their own paths according to their own wishes, just like my parents’ education approach to me. Of course, cultivating their abilities and means of survival is also necessary, so that they have the capacity to do what they want or at least take care of themselves. I still hold an “A-level driver’s license”, which my dad made me obtain before going to university, saying that in case I couldn’t find a job, I could work as a driver. Speaking of life, Xiaobo Wang said, “A person should be the master of his or her own life, not a commodity in someone else’s hands.” This is exactly what I mean.

**10. You have published important outcomes of research in many high-quality scientific journals both domestically and internationally. From your perspective, what are the advantages and disadvantages of Chinese Sci-Tech journals in terms of high-quality development?**

Domestic journals have many advantages, which primarily include the following points:

1. China has a large pool of scientific and technological talents that have provided abundant sources of papers and high-quality research outcomes for Sci-Tech journals.
2. China has been laying increasing emphasis on scientific and technological innovation as well as the development of Sci-Tech journals, with massive funds and resources put to support the development of Sci-Tech journals, indicating the country’s superiority in pooling together resources to accomplish major tasks.
3. Chinese journals have certain characteristics and advantages in certain fields that have shown distinctive development, such as materials and information science.

Despite advantages, there are still a few areas that need improvement for domestic journals.

1. Although the overall level of domestic journals is on the rise, there is generally still some room for improvement compared to international advanced journals, which is reflected not only in the impact factor but also in the recognition by researchers. For example, many researchers, including Chinese researchers, tend to contribute to well-established international prestigious journals, despite some Chinese journals already having high impact factors. This is primarily because these well-established journals have built a good reputation, and reputation matters. The impact factor involves citation and the number of published papers but cannot fully represent reputation. So how to enhance the reputation of domestic journals is the major task at present.
2. Chinese scientific journals are not international enough, which means we are not appealing enough to international researchers. One reason for this is that international researchers are not familiar enough with our journals. I still remember that at the start of the establishment of *Light*, Prof. Ping Koy Lam from the Australian National University told me that he thought it was good to publish his article in *Light* because the same issue also featured articles by Nobel laureates. So we need to increase internationalization and attract excellent papers from well-known international research groups.
3. There is still room for optimization of the review system and the editorial and publishing processes for Chinese Sci-Tech journals. It would be perfect if each paper could be sent to appropriate reviewers with relevant backgrounds. And it would be better if editors had a better knowledge of whether a scientific paper was well written or not. But for journal editors, especially part-time academic editors, time is limited, and the papers that they often deal with usually cover a wide range of research areas, which makes it quite challenging in this regard.

**11. What do you want to share with young scientists?**

There are many words of encouragement, but if I were to condense them into one word, it would probably be “perseverance”. Adversity often comes unexpectedly. Just when everything seemed perfect yesterday, it can be followed by a night of continuous rain today, and the day after, the sun might shine brightly again. This is the norm in academia and in life. Research is very pure, as it’s about exploring the unknown and gaining a sense of achievement from “mapping the uncharted territory”. But to preserve this purity, we must first ensure our own and our team’s survival, and that involves dealing with a bunch of troubles that can easily distract us from the purity. Our task is to stand firm, not forget our original intentions, and not let trivial matters blind us. But saying it is easy, doing it is difficult. Five or six years ago, on a sunny day when I was in a bad mood, I was on a train to work and passed by a large billboard advertising an education and training institution. Although the train was fast, I could see it clearly. It showed a photo of my former collaborator, the Australian young talent Alexander Argyros, smiling very genuinely and happily. That day, I started looking for jobs in companies, and I almost said goodbye to the academia when I received an offer. But I eventually couldn’t bear to leave the academia and stayed. I remember that day, I even had a little drink, toasting to the uncertainty of life, to the academia, to the optical setups that we had tried to align but sometimes just couldn’t get right, and to ourselves as researchers.

**Figure:**
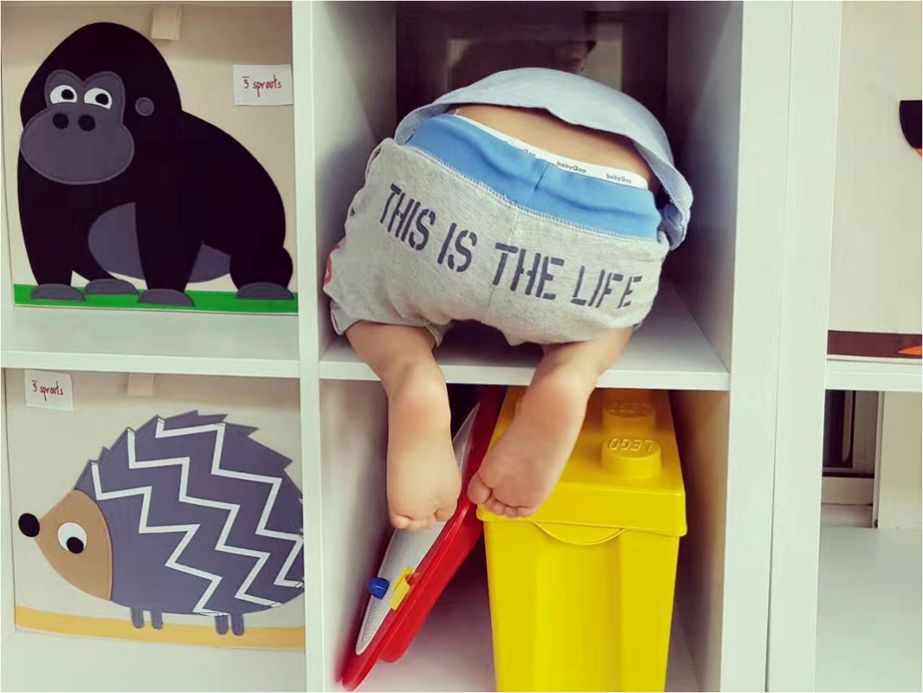
“This is the life, it goes up and down.” My eldest son once crawled into a cabinet compartment, and the words “This is the life” on his backside stood out, just like life itself. Sometimes, we climb up high and stand out, and other times, we fall to the ground and break into pieces
